# Evaluating Smoking Cessation Interventions and Cessation Rates in Cancer Patients: A Systematic Review and Meta-Analysis

**DOI:** 10.5402/2011/849023

**Published:** 2011-07-10

**Authors:** Smriti Nayan, Michael K. Gupta, Doron D. Sommer

**Affiliations:** Division of Otolaryngology, Head and Neck Surgery, McMaster University, 1200 Main Street West Hamilton, ON, Canada L8N 3Z5

## Abstract

*Background*. Tobacco smoking cessation interventions in the oncology population are an important part of comprehensive treatment plan. *Objectives*. To evaluate through a systematic review smoking cessation interventions and cessation rates in cancer patients. *Search Strategy*. The literature was searched using Medline, EMBASE, and the Cochrane Library (inception to November 2010) by three independent review authors. *Selection Criteria*. Studies were included if tobacco smoking cessation interventions were evaluated and patients were randomized to usual care or an intervention. The primary outcome measure was cessation rates. *Data Collection and Analysis*. Two authors extracted data independently for each paper, with disagreements resolved by consensus. *Main Results*. The systematic review found eight RCTs investigating smoking cessation interventions in the oncology patient population. Pooled relative risks were calculated from two groups of RCTs of smoking cessation interventions based on followup duration. In both groups, the pooled relative risk did not suggest a statistically significant improvement in tobacco cessation compared to usual care. *Conclusions*. Our review demonstrates that recent interventions in the last decade which are a combination of non-pharmacological and pharmacological approaches yield a statistically significant improvement in smoking cessation rates compared to usual care.

## 1. Introduction

Smoking tobacco not only predisposes to the development of disease, it increases disease severity and treatment failure rates. For these reasons, tobacco cessation is a critical part of the treatment of patients with cancer. Smoking cessation particularly benefits those patients who have smoking-related cancers such as head and neck or lung cancer and those who are diagnosed with curable disease [1]. 

Cigarette smoking and alcohol consumption are well-established risk factors for developing squamous cell carcinoma of the head and neck. Furthermore, a number of studies have revealed an association between tobacco carcinogens and the molecular progression of squamous cell carcinoma of the head and neck [2]. In countries with a high prevalence of smoking, approximately 90% of diagnoses of lung cancer are attributable to cigarette smoking. Furthermore, the increased incidence of lung cancer from smoking is proportional to the length and intensity of smoking history [3]. Smoking cessation before diagnosis reduces the risk of developing a primary tumour of all major histological types of lung carcinoma [3]. 

Prolonged tobacco smoking in cancer patients has many adverse effects during the oncology treatment plan as well. Studies with head and neck and lung cancer patients demonstrate that tobacco smoking reduces survival time [4] and increases the risk of a recurrence or a second primary tumor [4, 5]. Smoking also reduces the efficacy of radiotherapy in head and neck cancer patients, as smokers have a lower rate of complete response and poorer 2-year survival rate than nonsmokers and those who quit prior to treatment [6]. Tobacco smoking also exacerbates and prolongs radiotherapy induced complications such as mucositis, dry mouth, loss of taste, voice, impaired pulmonary function, wound healing, as well as tissue and bone necrosis [4, 7]. Despite these adverse health effects, 23% to 35% of head and neck cancer patients [5, 8–10] and 13% to 20% of lung cancer patients who smoked prior to diagnosis continue to do so after diagnosis [4]. Comorbid conditions such as depression, disease-related anxiety, and alcohol abuse often make cessation challenging.

The diagnosis of cancer allows an opportunity for patients to review and change their lifestyle habits [11, 12]. The health care setting is an ideal place to initiate cessation interventions with smokers [13]. The earliest descriptions of smoking cessation interventions in the health care setting are described in a summary report by Schwartz [14]. Interventions may be hospital-based, community-based, or based on individual counseling. Current smoking cessation interventions can be either pharmacological, nonpharmacological or a combination of both. Examples of effective approaches include identifying tobacco use in patients, motivating them to quit and supporting them to quit through brochures and pamphlets, counseling, pharmacotherapy as well as regular followup [2].

Despite the substantial benefits of tobacco cessation in cancer patients, there is still a relative paucity of data on how to best achieve cessation in this population. The aim of our study was to systematically review the literature to summarize tobacco cessation interventions for cancer patients and the associated smoking cessation rates as a consequence of these interventions.

## 2. Methods

This systematic review was performed in accordance with a predetermined protocol consisting of eligibility criteria, a search strategy, outcomes, and statistical analysis. Our primary aim was to perform a pooled analysis of smoking cessation rates if appropriate. 

### 2.1. Literature Search Strategy

The literature was searched using OVID Medline (1950 through November 2010), EMBASE (1980 to November 2010), and the Cochrane Library (Cochrane Database of Systematic Reviews (2010 Issue 12). We used similar strategies to search all databases. Relevant articles and abstracts were selected and reviewed, and the reference lists from these sources and recent review articles or meta-analyses were searched for additional publications. 

The literature search of the electronic databases combined disease specific keywords (cancer, neoplasm, and malignancy) with outcome specific keywords (tobacco cessation, smoking cessation, nicotine cessation, quit rates, patient education, and patient intervention) for the following study designs and publication types: retrospective studies, prospective cohort studies, randomized controlled trials (RCTs), systematic reviews, and meta-analyses. The literature search was not limited for study design or publication date to ensure all relevant published articles were captured. All three study authors performed a search using individual strategies.

### 2.2. Study Selection Criteria

The three authors reviewed the studies identified by the search strategies for relevance. Disagreements were resolved by consensus. Reasons for exclusion were noted. Articles were included in the systematic review of the evidence if they were fully published reports or abstracts of randomized controlled trials (RCTs) evaluating a tobacco smoking cessation intervention versus standard usual care in the adult smoking cancer population (>18 years of age). To be included, trials had to report cessation rates at followup. 

Articles were excluded if they were published in a language other than English, did not discuss a tobacco cessation intervention, discussed nontobacco products without separation of data, or were in the pediatric population.

### 2.3. Data Extraction

Relevant data was extracted from fully published reports by two independent review authors using prescribed tables. Any disagreement was resolved with discussion and consensus. Primary authors of included studies were contacted if further elaboration on data was needed [1, 13]. Where studies were duplicated, the larger data set was used for the analysis [13, 18].

### 2.4. Statistical Analysis

Statistical calculations were performed using the StatsDirect software (Chesire, UK). Given that we analyzed prospectively gathered data from randomized trials, we calculated a pooled relative risk. The relative risk was a ratio of the risk of tobacco cessation in the intervention group versus the risk of tobacco cessation in the control group. The relative risks were calculated where data was available by intention-to-treat analysis. 

Pooled relative risks were calculated using the Rothman-Boice type of Mantel-Haenszel method assuming fixed effects [15]. The random-effects model of pooled relative risks was calculated using the DerSimonian-Laird method [19]. The decision to use either a random effects model or a fixed effects model was based on calculation of heterogeneity of the data using an *I* ^2^ calculation. Where heterogeneity of the data was large, a random effects model was used. Confidence intervals of the pooled relative risk were calculated using the Greenland-Robin variance formula [16].

## 3. Results

### 3.1. Literature Search

**Figure 1:**
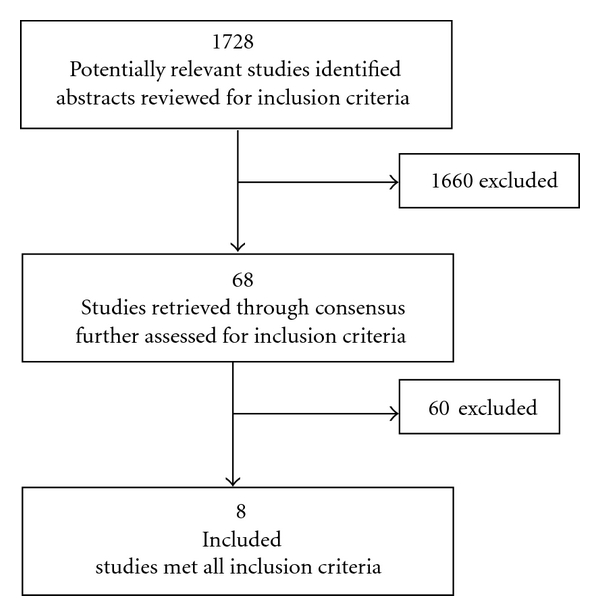
Flow diagram outlining our search strategy.

Eight studies [1, 4, 11, 13, 17, 20–22] were narrowed down through the search (Figure 1). All the studies identified were RCTs in the adult smoking population which described a tobacco smoking cessation intervention and smoking cessation rates.

### 3.2. Trial Characteristics

All published studies (Table 1) were RCTs, which were either single center [11, 13, 17, 21, 22] or multicenter [1, 4, 20]. There were a total of 1304 patients included. The included studies did not show significant differences in baseline characteristics of mean age and gender distribution except in studies that included only head and neck patients [11, 13] (Table 1). Smoking cessation interventions were delivered by the health care team. Interventions were nonpharmacological (cognitive behavioral therapy, self-help material, education modules, motivational interviewing), or pharmacological (nicotine replacement therapy or bupropion) (Table 2). All studies evaluated tobacco smoking.

### 3.3. Tobacco Cessation Rates and Followup

All the studies used self-reported rates of tobacco cessation. In addition, some studies used biochemical verification as well for smoking cessation. Two of the studies used breath testing of carbon monoxide (CO) [11, 21], two studies used urine cotinine levels [1, 11] and two studies used saliva cotinine levels [13, 17] where possible to confirm self-reported rates (Table 2). 

Cessation rates depending on randomization varied for each study at different followup times (Table 3). The studies were evaluated in two groups, one group with a shorter followup time, mean of 5 weeks [13, 17, 22] and the other group had a longer followup time of at least 6 months [1, 4, 11, 20]. In the second group, pooled rates were calculated from the 6 month abstinence rates in all studies except in one study which did not report a 6 month rate but only a 12 month rate [1]. Schnoll et al'.s study [21] was excluded from the longer followup group and analysis due to a purely pharmacological intervention compared to the other studies in the other group which were combination interventions.

**Figure 2:**
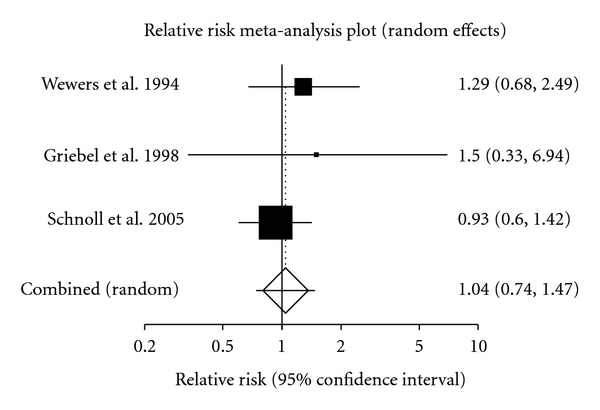
Meta-analysis plot looking at relative risk in the shorter followup group.

**Figure 3:**
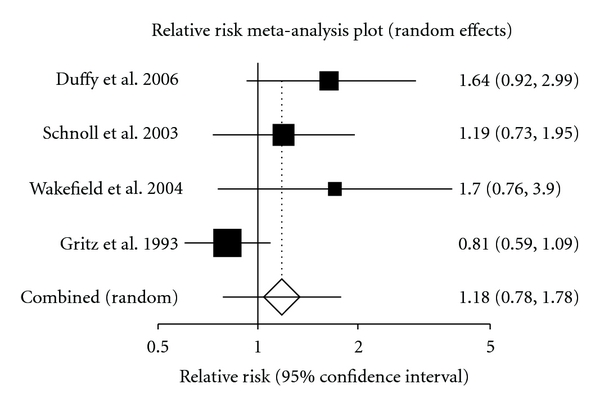
Evaluating the relative risk through random effects for the longer followup group.

For the purpose of pooling data, only self-reported data was pooled. When we examined the shorter followup group, the pooled relative risk was calculated to be 1.16 (95% CI = 0.80 to 1.76) in the short followup group (Figure 2). This was calculated using the fixed effects model. The longer followup group had a pooled relative risk of 1.19 (95% CI = 0.78 to 1.78) (Figure 3). The random effects model was used to calculate this pooled relative risk. When the oldest study was excluded form this series the result changed dramatically [1]. The pooled relative risk of the three most recent studies shows a result that is much more positive, with the relative risk achieving statistical significance. This pooled rate was calculated to be 1.42 (95% CI, 1.05 to 1.94).

## 4. Discussion

The systematic review identified eight studies which met inclusion criteria. We used a broad search strategy with multiple reviewers. When necessary, authors were contacted to provide additional data to ensure an accurate data set. The identified papers were methodologically sound with prospectively gathered data, randomized study populations, and suitable control groups. 

All eight studies had slightly different smoking cessation treatment plans. Only one study [21] looked at a purely pharmacological approach to tobacco cessation while three studies had only a nonpharmacological approach [1, 13, 17]. Four studies [4, 11, 20, 22] had a combination approach of nicotine replacement therapy, counseling and/or cognitive behavioral study. Duffy et al. [20] looked at the combination of cognitive behavioral therapy in addition to bupropion and nicotine replacement therapy. Comparison of the confidence intervals of the calculated relative risks of the studies does not demonstrate a significant difference in tobacco smoking cessation rates between any of the different types of intervention.

Similar to other systematic reviews, our analysis comprised of a relatively high heterogeneity of treatments and patient groups. This type of heterogeneity is common and should be incorporated into systematic reviews [19]. We incorporated this heterogeneity when present using random effects models. 

The studies were divided into two groups—short followup time (mean 5 weeks) and longer followup time (6 months). While comparing the pooled relative risks of the two groups, it does not demonstrate a significant difference in the cessation rates despite the fact that a longer followup time would tend to represent better cessation rates. However, considering the shorter followup times, it is difficult to state whether at 6 months, this group would continue to have the same cessation rates. 

When we examined the longer followup group, four studies were included [1, 4, 11, 20]. The original study was performed in the early 1990 [1]. This study examined a group of 186 head and neck cancer patients and utilized a nonpharmacological counseling-based approach. If this original study is excluded from the analysis, this long-term followup group does appear to yield a statistically significant effect due to a multi-modal cessation intervention. Notably, these other three studies [4, 11, 20] were performed in the most recent decade and examined 705 patients with diverse oncologic diagnoses. Furthermore, these three studies all included a pharmacological approach in addition to nonpharmacological intervention. These differences may account for the reason that the pooling most recent studies reaches a statistically significant effect for smoking cessation intervention. 

In addition, the setting in which the cessation methods were offered are also different. Some studies counseled patients on smoking cessation during a postoperative hospital admission, while other interventions were provided in the clinics. Comparing the cessation rates of the individual studies does not demonstrate a significant difference in the cessation patterns based on setting. 

Not all studies performed biochemical verification of tobacco cessation. Consequently, our pooled rates were calculated for self-reported tobacco cessation. It is unlikely the self-reported rates would bias our review towards a negative result. 

When all studies are analyzed in the short and long-term, there was no statistical to a tobacco smoking cessation intervention in addition to usual care. However, when examining the effect of a multifaceted approach (pharmacological and nonpharmacological) to smoking cessation, the analysis suggested a more positive result.

## 5. Conclusion

There are few RCTs evaluating smoking cessation interventions. Our review does not demonstrate a significant difference in tobacco smoking cessation rates through these interventions. The data does, however, suggest that the combination of both pharmacological and nonpharmacological approaches may be more successful at achieving tobacco cessation. 

Tobacco cessation is a formidable challenge in this complex patient population. Collaboration within the health-care team is paramount in implementing a smoking cessation intervention. The significant benefits of tobacco cessation demand the oncology team continue to explore and investigate novel and known methods to help patients become tobacco-free.

## Figures and Tables

**Table 1:** Trial characteristics.

Author year (Ref)	Study type	Study dates	Single-center or multicenter	No. of pts	Patient characteristics				
					Mean age ± SD (range) (yrs)	Gender		Mean followup (mos)	Primary site of malignancy (% no. of pts)
						M (no.)	F (no.)		
Schnoll et al. 2003 [4]	RCT	NR	Single	74	57 ± 11.4	38	36	3	Lung (69), Head and Neck (31)
Wakefield et al. 2004 [11]	RCT	2002–2008	Single	246	54.8 ± NR	128	118	6	Lung or Head and Neck (32.2), Breast (21), Prostate (15), Lymphoma (9), Colorectal (5), Kidney, pancreas, liver (4), GU (3), Esophageal (3), Other or multiple primaries (5)
Gritz et al. 2006 [12]	RCT	1999–2001	Single	137	I: 52.6 ± 13.8, U: 51.9 ± 11.5	85	52	6	Lung (12), Head and neck (17), Bladder (2), Breast (13), Prostate (9), Colorectal (10), Leukemia (10), Lymphoma (15), Testicle (4), Other (8)
Wewers et al. 1994 [13]	RCT	2000–2003	Multi (4 hospitals)	184	57 ± 9.9	155	29	6	Head and Neck: larynx (33), oropharynx/hypopharynx (30), oral cavity/other (37)
Gritz et al. 1993 [1]	RCT	NR	Multi (10 clinics)	186	C: 57.8 ± 9.5, NC: 59.5 ± 9.5	137	49	12	C: buccal cavity 59(52.7%), pharynx 6 (5.4%), larynx 47 (42.0%); NC: buccal cavity 58.3 (42%), pharynx 5 (6.9%), larynx 25 (34.7%)
Rothman and Greenland 1998 [15]	RCT	NR	Single	30	I: 56.4 ± 13.6, U: 53.2 ± 13.3	10	20	5 weeks	Heak and Neck (83.3), Breast cancer (6.7), Prostate cancer (6.7), Cervical cancer (3.3)
Greenland and Robins 1985 [16]	RCT	NR	Single	28	I: 50.2 ± 12.4, U: 51.9 I: 50.2 ± 10.5	14	16	1.5	Gynecologic (6.21), Breast (5.18), Gastrointestinal (4.14), Thoracic (4.14), Urologic (4.14), Neurologic (4.14), Head and Neck (2.7)
Griebel et al. 1998 [17]	RCT	NR	Single	109	I: 58.7 ± 9, U: 57.7 ± 10.1	59	50	1 and 3	Head and Neck (69), Lung (31)

F, female; I, intervention; M, male; mos, months; no., number; NR, not reported; NS, not significant; pts, patients; ref, reference; U, usual care; yrs, years, C: completers, NC: non-completers.

**Table 2:** Tobacco smoking cessation intervention characteristics.

Author, year (Ref)	No. of pts	Smoking type	Current smokers	Cigarettes smoked/day ± SD	Randomization characteristics					
					Usual care (no. of pts)	Intervention (no. of pts)	Cessation intervention			Confirmation of non-smoking
							Setting	Non-pharmacological	Pharmacological	
Schnoll et al. 2003 [4]	74	Tobacco	74^a^	NR				counseling	nicotine replacement therapy	Self-report
Wakefield et al. 2004 [11]	246	Tobacco	246	17.5 ± 9.6	132	118		none	Nicotine replacement therapy, buproprion	Self report and breath CO
Gritz et al. 2006 [12]	137	Tobacco	135	I: 21.7 ± 12.1, U: 21.4 ± 10.9	63	74		motivational interviewing, smoking cessation booklets, family advice to quit	nicotine replacement therapy	Self-report, breath CO and urine continine levels
Wewers et al. 1994 [13]	184	Tobacco	136	NR	91	93		Cognitive behavioral therapy	nicotine replacement therapy and buproprion	Self-report
Gritz et al. 1993 [1]	186	Tobacco	96	C: 24.0 ± 12.4, U: 21.4 ± 11.3	46	50	clinic	Counseling, booklets	none	Self-report, urine continine levels^e^
Rothman and Greenland 1998 [15]	30	Tobacco	30	I: 19.1 ± 7.7, U: 23.4 ± 9.6	16	14	Post-operative, in-hospital	Counseling, cessation booklets,	none	Self-report and saliva continine levels^b,c^
Greenland and Robins 1985 [16]	28	Tobacco	28	I: 24.7 ± 14.5, U: 27.0 ± 19.8	18	18	In-hospital, during hospitalization, pre-operative	Counseling, booklets	none	Self-report and saliva continine levels^d^
Griebel et al. 1998 [17]	109	Tobacco	108	I: 19.2 ± 12.3, U: 17.5 ± 11.2	57	52		Counseling	Nicotine replacement therapy	Self-reported

I, intervention; U, usual care, NR: not reported; CO: carbon monoxide

^a^: smoking within 6 months of diagnosis

^b^: saliva continine level of ≤10.0 ng/mL

^c^: 1 patient declined saliva continine level

^d^: saliva continine level of ≤14.0 ng/mL

^e^: urine continine level of ≤50 ng/mL.

**Table 3:** Summary of tobacco smoking cessation rates.

Study	Intervention group		Usual care group	
	Total no. of pts	No. patients who ceased smoking (%)	Total no. of pts.	No. of pts who ceased smoking (%)
*Long followup (mean 6 mo.) *				
Duffy et al. 2006 [20]	62	23	53	12
Schnoll et al. 2003 [4]	215	30	214	25
Wakefield et al. 2004 [11]	74	14	63	7
Gritz et al. 1993 [1]	50	29	46	33
Schnoll et al. 2010 [21]	114	21	132	23

*Short followup (5-6 weeks)*				
Wewers et al. 1994 [13]	14	9	16	8
Griebel et al. 1998 [17]	18	3	18	2
Schnoll et al. 2005 [22]	52	22	57	26

## Abbreviations

RCT: Randomized control trial.
